# Fast, whole-heart, free-breathing 3D T2 mapping at 3T with application to myocardial edema imaging

**DOI:** 10.1186/1532-429X-17-S1-Q125

**Published:** 2015-02-03

**Authors:** Hsin-Jung Yang, Jianing Pang, Behzad Sharif, Avinash Kali, Xiaoming Bi, Ivan Cokic, Debiao Li, Rohan Dharmakumar

**Affiliations:** 1 Cedars Sinai Medical Center, Los Angeles, CA, USA; 2 Siemens Healthcare, Los angeles, CA, USA

## Background

Cardiac MRI (CMR) T2 mapping is a proven method for myocardial edema detection. However, the current approach requires multiple breath-holds and can take nearly 10-15 minutes to complete. Faster acquisitions could potentially improve patient comfort and cost-effectiveness of CMR exams. the objecttive of this study is to develop and test a free-breathing, three-dimensional, cardiac MR approach which can yield fast, accurate, T_2_ maps of the whole left ventricle at 3T.

## Methods

We developed an ECG-triggered, free-breathing, T_2_-prepared, three-dimensional gradient-echo acquisitions with different echo times (0, 24, 55 ms) with near perfect navigator efficiency on a clinical 3T system. The proposed approach was tested and validated in ex-vivo porcine hearts, healthy volunteers and canines with reperfused acute myocardial infarction (rAMI). On the basis of the navigator signals, images were corrected for respiratory motion and were fit to a mono-exponential function to derive T_2_ maps of the whole left-ventricular myocardium.

## Results

Ex-vivo myocardial T_2_ values of the proposed approach (3D FB MoCo) were not different from standard 2D approaches (all p<0.05): 48.7±0.9 ms (3D FB MoCo) vs. 48.2±0.6 ms (2D spin echo) and 47.5±0.8 ms (2D T_2_-prepared bSSFP (T_2_-prep bSSFP)). In healthy volunteers, compared to 3D FB MoCo and 2D BH, myocardial T_2_ maps, 3D FB Non-MoCo T_2_ myocardial maps showed longer T_2_ values (p<0.05), larger coefficient-of-variations (COV) in T_2_ (p<0.05), and lower image quality (p<0.05). Conversely, the mean and COV in myocardial T_2_ and image quality of 2D BH and 3D FB MoCo T_2_ were not different (p=0.99, p=0.74, p=0.14, respectively). In canines with rAMI, edema volumes measured from 2D BH and 3D FB MoCo T_2_ maps were closely correlated (both R^2^ = 0.97 and p<0.05). In Bland-Altman analysis, mean T_2_ of edematous and remote zones and edema volumes were within the limits of agreement (bias in T_2_ = 0.4 ms and edema volume = 0.9%).

## Conclusions

The proposed free-breathing, three-dimensional T_2_ mapping approach at 3T enabled whole-heart acquisitions within 5 minutes with an accuracy in T_2_ not different from that of the state-of-the-art breath-held T_2_ mapping approach.

## Funding

This work was supported in part by National Heart, Lung, and Blood Institute HL091989 (R.D.).The content is solely the responsibility of the authors and does not necessarily represent the official views of the National Heart, Lung, And Blood Institute or the National Institutes of Health.

**Figure 1:**
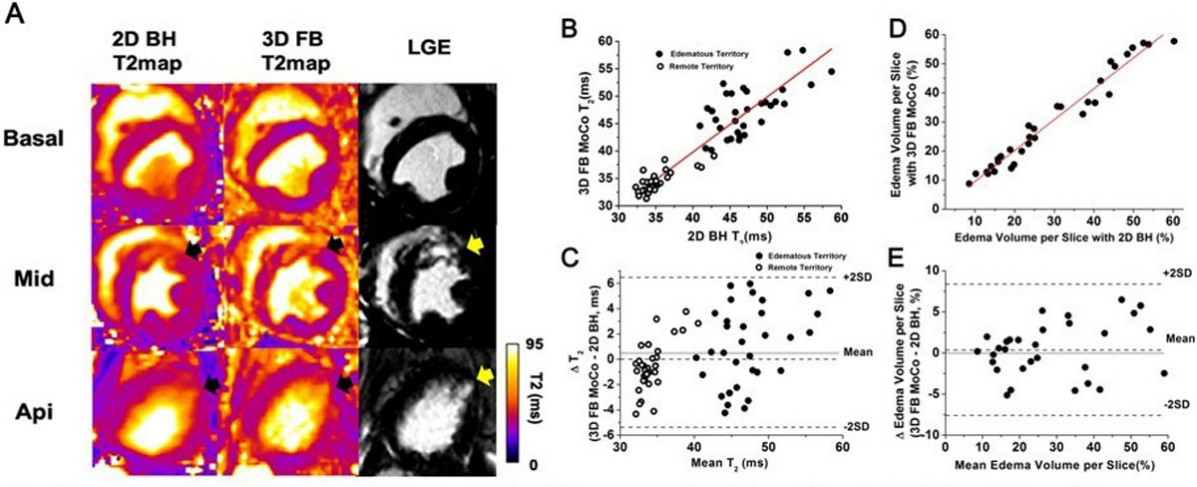
Representative short-axis images obtained from a canine (A) and the statistical relations between 2D BH and 3D MoCo for T2 and edema volume across all animals on day 4 post myocardial infarction (B-E). (A) Slice-matched T2 maps from 2D BH and 3D FB MoCo, as well as LGE images obtained from a canine at the basal, mid ventricle (Mid) and apical sections are shown. Note the close correspondence between hyperintense regions in LGE images (yellow arrows). Also not that the 2D BH images are blurrier due to cardiac motion (from single-shot acquisitions in the presence of high heart rates in canines with recent infarction), which is not the case with the proposed approach. (B) Linear regression analysis between T2 values (edematous territories and remote myocardium) of slice-matched 3D FB MoCo and 2d BH acquisitions and the corresponding Bland-Altman analysis are shown in panels B and C, respectively. Linear regression: y = 0.9 x – 3.4, where y = T2 from 2D BH acquisitions, with R2 = 0.88, p<0.05. Linear regression analysis between slice-matched 3D FB MoCO and 2D BH acquisions to Edema volume and the corresponding Bland-Altman analysis are shown in panels D and E, respectively. Linear regression: y1 = 1.1 x1 -2.4, where y1 = Edema volume from 3D FB MoCO and x1 = Edema volume from 2D BH acquisitions, with R2 = 0.96, p<0.05.

